# Optical Coherence Tomography Angiography: Considerations Regarding Diagnostic Parameters

**DOI:** 10.18502/jovr.v17i3.11567

**Published:** 2022-08-15

**Authors:** Touka Banaee

**Affiliations:** ^1^Department of Ophthalmology and Visual Sciences, University of Texas Medical Branch, Galveston, Texas, USA

In this issue of *Journal of Ophthalmic and Vision Research* (*JOVR*), two articles are published addressing changes in optical coherence tomography angiography (OCTA) parameters after acute changes in ocular perfusion pressure (OPP). The first article by Ashraf Khorasani et al^[1]^ is a study on changes in vascular density (VD) of the macula and optic nerve after an acute rise in intraocular pressure (IOP), and the second paper is a case report by Mirshahi et al^[2]^ describing OCTA changes in acute systemic hypertension.

Studies on hemodynamics of the retina and optic nerve microvasculature is an old but still open area of research. With technologies evolving over time, a variety of diagnostic modalities have been used for detection and documentation of blood flow and vascular diameter, including fluorescein angiography, color Doppler ultrasonography, Doppler velocimetry, laser speckle flowgraphy, and Doppler OCT.^[3,4,5,6,7]^

OCTA is a relatively new technology used for extraction of blood vessels on OCT images by detecting movement of blood. This technology has been extensively used to evaluate circulation in different retinal vascular layers (superficial vascular plexus, SVP and deep vascular plexus, DVP) and choriocapillaris. Evaluation of the peripapillary vascular network and optic disc vessels with OCTA has also become an active area of research in glaucoma.

While the retina and optic nerve provide a unique target for *in vivo* evaluation of microvasculature, results of studies on this intricate system may not always corroborate with each other. This is due to the complex interplay between blood pressure, intraocular pressure, and retinal/optic nerve autoregulation, where subtle differences in study parameters can affect the response of blood vessels and impact the study results. The difference between ocular arterial pressure and intraocular pressure (IOP) determines the ocular perfusion pressure (OPP). As the ocular arterial pressure cannot be easily measured, brachial artery pressure has been used as its surrogate in most studies, although some corrections may be needed in the upright position.^[8]^ Autoregulation of blood flow is another factor that affects hemodynamics of ocular tissues, especially the retina and optic nerve, and needs to be considered in these types of studies. As autoregulation may need some time to fully take place, timing of measurements after a stimulus/event also seems to be important.^[9]^

Concerning the wide availability of OCTA machines, this modality has been extensively used to evaluate changes in retinal/optic nerve circulation under different conditions. There are two quantitative measures in OCTA that are mostly used to describe changes in retinal and optic nerve vasculature: flux and VD. Flux is the intensity of flow signals while VD is the proportion of flow signals on the en-face OCTA image.^[10]^ Although not as accurate as methods that directly measure blood flow and vessel diameter such as laser speckle flowgraphy and Doppler OCT, VD in OCTA can be considered a useful clinical parameter to reflect changes in blood flow and vascular diameter.^[11]^ When using this parameter to study ocular hemodynamics, some important points need to be kept in mind:

OCTA detects blood flow above a certain threshold; above that threshold, the OCTA signal cannot reflect flow rate. Hence, subtle changes in blood flow may not be detected by VD. At the same time, it is very sensitive to changes in small vessels diameter, even below its resolution.^[11]^

VD also depends on the total length and diameter of vessels and will be affected by loss of small vessels in diseases such as diabetic retinopathy and retinal vein occlusions.

VD is the sum of flow signals in an image and includes flow from all vessel diameters in the specified area, therefore the study area will define which vessel sizes are included. For example, VD in the parafoveal area will be a better representative of small vessels as compared to VD of a whole 3 × 3 or 6 × 6 OCTA picture.

VD is affected by multiple parameters in addition to blood flow, including low signal strength. So, considering media quality (especially presence of cataracts) and excluding low signal strength images is crucial to ensure validity of data.^[12]^

Previous studies have used various methods to increase IOP in human eyes, including dark room prone positioning in eyes prone to angle closure^[13]^ and application of a suction cup.^[6]^ There are also studies investigating changes in vasculature during IOP rise after laser iridoplasty or intravitreal injections.[9, 14] The response of the vascular structure may be different with acute rises in IOP such as when a suction cup is applied, or after intravitreal injections as compared to slower increases in IOP as happens after laser iridoplasty or dark room prone positioning.^[13]^ Therefore, results of different studies may not be readily comparable.

The article by Ashraf Khorasani et al is an interventional study, evaluating the effect of acute IOP rise by application of a suction cup on VD in the DVP and SVP, the peripapillary region and inside the disc in 12 healthy individuals and 12 patients with mild-moderate NPDR. They achieved a mean IOP elevation of 13.93 ± 3.41 mmHg and found that VD in both SVP and DCP decreased in healthy individuals, while in diabetic patients there was a significant reduction of VD only in the DVP. In both groups, VD inside the disc was decreased significantly, while changes in the radial peripapillary capillaries was not significant.

The two groups had different baseline OCTA parameters, which can be explained by the difference in age and the presence of diabetes with its associated effect on ocular vessels.[15, 16] As the DVP is considered to be mostly on the venous side of the retinal circulation,^[17]^ it may be more affected by changes in intraocular pressure. On the other hand, older patients with diabetes may have stiffer arteries that are less affected by changes in perfusion pressure; hence the SVP, which is closer to the arteries, may be less affected by a moderate amount of IOP rise. In this study, changes in VD in response to IOP rise was not different between the two groups. These results should be interpreted reservedly, as the study may have been underpowered to detect such a difference because of small sample size.

Another factor to consider is that stress during placement of the suction cup may cause blood pressure to rise and may partially mitigate the effect of IOP rise by increasing ocular prefusion pressure. Also, the degree of blood pressure rise may differ based on age and presence of diabetes. Although blood pressure has been reported to be steady in healthy subjects during application of a suction cup in a prior study,^[6]^ it would have been informative if blood pressure readings at the time of IOP rise were available.

The different response of the optic disc vessels, originating from choroidal arteries, and the peripapillary radial vessels which originate from the central retinal artery, to acute IOP rise can be a confirmation of the previously known fact of different pressures within the retinal and choroidal systems.^[18]^ Prior studies have not reported a different response by radial peripapillary capillaries and vessels inside the disc after an acute rise in IOP by intraocular injections.[9, 19] As stated before, the complex interplay of BP, IOP, retinal/optic disc autoregulation and the timing of the measurements after IOP rise affects the results of studies and makes standardization and comparisons difficult.

Although a small study with its limitations, this paper adds to the body of the evidence in this field and the authors should be congratulated for their work.

The second article by Mirshahi et al is a case report describing OCTA findings in acute hypertensive retinopathy. Acute hypertensive retinopathy can cause various tissue reactions alongside the increase in ocular perfusion pressure: vascular contraction due to autoregulation, vascular leakage, and thrombosis.^[20]^ Although an increase in BP increases OPP, autoregulation counteracts it and with vascular leakage and thrombosis happening in acute cases, there may be a decrease in blood flow and ischemia in OCTA contrary to what is expected from an increase in OPP. Ischemia is especially evident in the B scan OCTA images of this case as parafoveal acute middle maculopathy (hyper-reflectivity of the outer plexiform layer), coinciding with locations where capillary loss is visible in the SVP and DVP. Depth-enhanced visualization of blood vessels, a characteristic of OCTA, has enabled better visualization of retinal capillary loss. Better visualization of ischemia in the choriocapillaris in comparison to FA can be attributed to the longer wavelength of infrared light used in OCTA that penetrates better through the RPE as compared to the blue light of fluorescein angiography (FA).

These two articles remind us of the fact that in biological systems, the response to stimuli may not follow gross physical rules and need to be interpreted taking into account variability of intricate biological responses which may not always be simple to elucidate.
